# Four New Dicaffeoylquinic Acid Derivatives from Glasswort (*Salicornia herbacea* L.) and Their Antioxidative Activity

**DOI:** 10.3390/molecules21081097

**Published:** 2016-08-20

**Authors:** Jeong-Yong Cho, Jin Young Kim, Yu Geon Lee, Hyoung Jae Lee, Hyun Jeong Shim, Ji Hye Lee, Seon-Jae Kim, Kyung-Sik Ham, Jae-Hak Moon

**Affiliations:** 1 Department of Food Science and Technology and Functional Food Research Center, BK21 Plus Program, and Chonnam National University, Gwangju 61186, Korea; jyongcho@mokpo.ac.kr (J.-Y.C.); jykim78@korea.kr (J.Y.K); ugun2@naver.com (Y.G.L.); leehj79@port.kobe-u.ac.jp (H.J.L.); shj1004v@naver.com (H.J.S.); psk9896@naver.com (J.H.L.); 2 Department of Food Engineering and Solar Salt Research Center, Mokpo National University, Jeonnam 58554, Korea; ksham@mokpo.ac.kr; 3 Jeollanam-Do Institute of Health and Environment, Jeonnam 58568, Korea; 4 Department of Marine Bio Food Science, Chonnam National University, Yeosu 59626, Korea; foodkims@jnu.ac.kr

**Keywords:** *Salicornia herbacea*, caffeoylquinic acid derivatives, antioxidant, radical-scavenging activity, blood plasma oxidation

## Abstract

Four new dicaffeoylquinic acid derivatives and two known 3-caffeoylquinic acid derivatives were isolated from methanol extracts using the aerial parts of *Salicornia herbacea*. The four new dicaffeoylquinic acid derivatives were established as 3-caffeoyl-5-dihydrocaffeoylquinic acid, 3-caffeoyl-5-dihydrocaffeoylquinic acid methyl ester, 3-caffeoyl-4-dihydrocaffeoylquinic acid methyl ester, and 3,5-di-dihydrocaffeoylquinic acid methyl ester. Their chemical structures were determined by nuclear magnetic resonance and electrospray ionization-mass spectroscopy (LC-ESI-MS). In addition, the presence of dicaffeoylquinic acid derivatives in this plant was reconfirmed by LC-ESI-MS/MS analysis. The isolated compounds strongly scavenged 1,1-diphenyl-2-picrylhydrazyl radicals and inhibited cholesteryl ester hydroperoxide formation during rat blood plasma oxidation induced by copper ions. These results indicate that the caffeoylquinic acid derivatives may partially contribute to the antioxidative effect of *S. herbacea*.

## 1. Introduction

Excessive reactive oxygen species cause oxidative damage in the body and are often the result of various human diseases including atherosclerosis, cancer, and aging [1]. Many studies have indicated that intake of foods such as vegetables and fruits containing plenty of antioxidants can reduce the risk of various diseases such as cardiovascular disease and cancer [2]. Therefore, the clinical importance of antioxidant-rich foods has received considerable attention [3,4].

Halophytes, which are grown in a saline environment, are regarded as a potentially useful medicinal and food source [5,6]. Halophytes are constantly exposed to salt-triggered oxidative stress. Consequently, these plants synthesize and accumulate various secondary metabolites including antioxidative phenolics and flavonoids with multiple biochemical defensive capabilities to maintain ion homeostasis and protect their cell functions in response to saline stress [7]. The compounds act as excellent antioxidants to prevent various diseases such as atherosclerosis, cancer, and aging induced by excessive oxidative stress in humans [6]. Therefore, halophytes are considered to be beneficial in both medicinal and food related applications.

*Salicornia herbacea* L. (Chenopodiacea, glasswort) is commonly utilized as a food resource and is generally consumed in a variety of ways, including as a raw vegetable, in salads or in fermented foods in both Korea and Europe [8,9,10]. Glasswort has been reported to show various biological effects including antioxidant [10], anticancer [11], antidiabetic [12], and immunomodulatory [13] activities. Chemical constituents such as sterols [14], caffeoylqunic acid derivatives [15], flavonoid derivatives [16], triterpenoid saponins [17], and pentadecylferulate [18] have been found in glasswort. In addition, in our previous study, dicaffeoylquinic acid derivatives and flavonoid glucosides in this plant were isolated and identified as antioxidative compounds [19]. Nevertheless, the presence of other unidentified antioxidative compounds in glasswort was implied by the results of our studies. Therefore, we performed further studies to isolate and identify the antioxidative compounds in glasswort using a guided 1,1-diphenyl-2-picrylhydrazyl (DPPH) radical-scavenging assay.

In this study, we describe the isolation and structural elucidation of four new and two known caffeoylquinic acid derivatives from glasswort as well as their antioxidative activities.

## 2. Results and Discussion

### 2.1. Isolation and Structural Determination of Antioxidative Compounds

**Figure 1:**
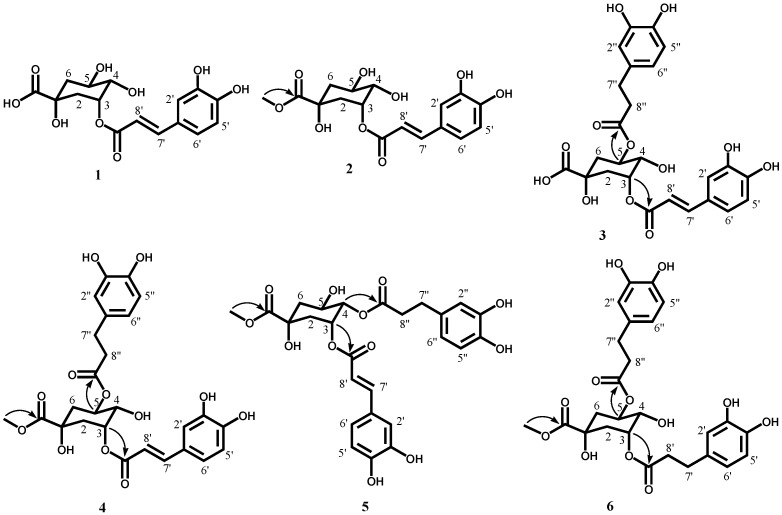
Structure of the isolated compounds and important HMBC correlations (arrows).

Six antioxidative compounds were isolated and identified from the ethyl acetate (EtOAc) fraction obtained after the partitioning of glasswort methanol (MeOH) extract by a guided DPPH radical-scavenging assay. Of these, two known compounds were identified as 3-caffeoylquinic acid (chlorogenic acid, **1**) and 3-caffeoylquinic acid methyl ester (methyl chlorogenate, **2**) [20] based on NMR and MS spectroscopic data (Figure 1).

The molecular formula for **3** was determined to be C_25_H_26_O_12_ (MW 518) by negative HRESI-MS data (*m/z* 517.1334 [M − H]^–^). The ^1^H NMR spectrum of **3** was also closely related to that of **1**. Here, the proton signals of the dihydrocaffeic acid moiety related to the tri-substituted aromatic ring protons at δ 6.67 (H-5″), 6.66 (H-2″), and 6.54 (H-6″) and two methylene protons at δ 2.79 (H-7″) and 2.60 (H-8″) were observed (Table 1). These results suggested that **3** consists of dihydrocaffeic acid, caffeic acid, and quinic acid. From the ^13^C-NMR spectrum, the results were further supported by the presence of 25 carbon signals for dicaffeoylquinic acid including three carboxylic carbon signals at δ 175.9 (C-7), 174.2 (C-9″), and 169.0 (C-9′) (Table 2). The proton signals of quinic acid including two methylenes at δ 2.11–2.27 (H-2, 6) and three oxygenated methines at δ 5.29 (H-5), 3.88 (H-4), and 5.37 (H-3) were detected in the ^1^H-NMR spectrum. The H-5 at δ 5.29 (1H, m) was downfield shifted by 1.16 ppm when compared to the ^1^H-NMR spectrum of **1**, suggesting that two of the three oxygenated methine groups in quinic acid were conjugated with dihydrocaffeic acid and caffeic acid. The quinic acid moiety was assigned based on their multiplicity and coupling patterns in the ^1^H NMR spectrum and the proton–proton correlations in the correlation spectroscopy (^1^H-^1^H COSY) spectrum. In the Overhauser effect (NOE) experiments for **3**, the signals for H-2 at δ 2.27 and 2.14 and H-4 at δ 3.88 were enhanced by irradiation of H-3 at δ 5.37. The signal for H-6 at δ 2.07 was also enhanced by irradiation of H-5 at δ 5.29. In addition, the signals for H-3 at δ 5.37, H-2 at δ 2.27, H-5 at δ 5.29, and H-6 at δ 2.11 were enhanced by irradiation of H-4 at δ 3.88. These data indicated that H-2 at δ 2.27, H-4 at δ 3.88, H-5 at δ 5.29, and H-6 at δ 2.07 were axial positions while H-2 at δ 2.14, H-3 at δ 5.37, and H-6 at 2.11 were equatorial positions. The connectivity of **3** was further confirmed by heteronuclear single quantum correlation (HSQC) and heteronuclear multiple-bond correlation (HMBC) experiments. The HMBC correlations (arrows) of δ 5.37 (H-3) to δ 169.0 (C-9′) and δ 5.29 (H-5) and δ 174.2 (C-9″) indicated that caffeic acid and dihydrocaffeic acid was esterified respectively with the C-3 and C-5 of quinic acid (Figure 1). Consequently, the structure of **3** was unambiguously determined to be 3-caffeoyl-5-dihydrocaffeoylquinic acid, which is a new compound (Figure 1). 

The molecular formula of **4** was determined to be C_26_H_28_O_12_ (MW 532) by negative HRESI-MS data (*m/z* 531.1500 [M − H]^–^). The ^1^H-NMR spectrum of **4** was closely related to that of **3**, except for a methoxyl group (δ 3.72) (Table 1). These results were also supported by the presence of 26 carbon signals assignable to dicaffeoylquinic acid coupled with a methoxyl group including three carboxylic carbons at δ 175.9 (C-7), 174.0 (C-9″), and 169.0 (C-9′) and a methoxyl carbon at δ 53.1 (-OCH_3_) detected in the ^13^C-NMR spectrum (Table 2). Based on the spectroscopic data from MS and ^1^H-NMR, **4** was proposed to be 3-caffeoyl-5-dihydrocaffeoylquinic acid methyl ester. The quinic acid moiety was assigned by ^1^H-^1^H COSY and NOE experiments, although the axial/equatorial features of H-6 at δ 2.11 could not be distinguished because the proton signals of H-6ax and H-6eq were overlapped. To the best of our knowledge, this compound has not been previously reported in nature. Therefore, the actual structure of **4** was determined by HSQC, ^1^H-^1^H COSY, and HMBC experiments. From the results of 2D-NMR spectra, **4** was determined to have the same structure as **3** except for the presence of a methoxyl group. In particular, a cross peak (arrow) between δ 3.72 (-OCH_3_) and δ 175.9 (C-7) was detected in the HMBC spectrum (Figure 1), indicating that the methyl group was esterified with the C-7 of quinic acid. Therefore, compound **4** was determined to be 3-caffeoyl-5-dihydrocaffeoylquinic acid methyl ester (Figure 1).

The molecular formula of **5** was determined to be C_26_H_28_O_12_ (MW 532) by negative HRESI-MS data (*m/z* 531.1507 [M − H]^–^). The ^1^H- and ^13^C NMR spectra of **5** were closely related to those of **4**, except for different chemical shifts for the quinic acid moiety (Table 1 and Table 2). In particular, the H-4 at δ 5.02 (1H, dd, *J* = 7.5, 3.5 Hz) was shifted downfield by 1.5 ppm and the H-5 at δ 4.26 (1H, m) was shifted upfield by 0.99 ppm when compared to the ^1^H NMR spectrum of **4**, suggesting that dihydrocaffeic acid is attached to the C-4 of 3-caffeoylquinic acid methyl ester. The contiguous protonated carbons (C-2–C-6) of the quinic acid moiety were assigned based on their proton–proton correlations detected in the ^1^H-^1^H COSY spectrum. In particular, a cross peak (arrow) between δ 5.02 (H-4) and δ 174.2 (C-9″) was observed in the HMBC spectrum (Figure 1), indicating that the dihydrocaffeic acid was esterified with the C-4 of quinic acid. Therefore, compound **5** was determined to be 3-caffeoyl-4-dihydrocaffeoylquinic acid methyl ester, which is also a new compound (Figure 1).

The molecular formula of **6** was determined to be C_26_H_30_O_12_ (MW 534) by negative HRESI-MS data (*m/z* 533.1655 [M − H]^–^). The ^1^H- and ^13^C-NMR spectra of **6** were closely related to those of **4**. However, the olefinic carbon proton signals for the caffeic acid in **4** were not observed. When the ^1^H- and ^13^C-NMR spectra of **6** were compared to those of **4**, a partial structure assignable to dihydrocaffeoylquinic acid was confirmed, similar to **4**, and the presence of a dihydrocaffeic acid other than caffeic acid, which was a partial structure of **4**, was suggested. That is, the dihydrocaffeic acid was assigned by the presence of proton signals including tri-substituted aromatic ring protons at δ 6.67 (H-5′), 6.66 (H-2′), and 6.55 (H-6′) and 2 methylene protons at δ 2.79 (H-7′) and 2.60 (H-8′) in the ^1^H-NMR spectrum (Table 1). The ^1^H-NMR data was also supported by the ^13^C-NMR spectrum (Table 2). From the MS and 1D-NMR spectra, **6** was proposed to be di-dihydrocaffeoylquinic acid methyl ester. In particular, correlations (arrow) from δ 5.18 (H-3, 5) to δ 174.6 (C-9′) and 173.9 (C-9″) were observed in the HMBC spectrum (Figure 1), indicating that two dihydrocaffeic acids are esterified, respectively, with the C-3 and C-5 of quinic acid methyl ester. Therefore, compound **6** was determined to be 3,5-di-dihydrocaffeoylquinic acid methyl ester, a new compound (Figure 1).

### 2.2. Qualification and Quantitation of ***3**–**6*** in the Aerial Parts of S. herbacae

**Figure 2:**
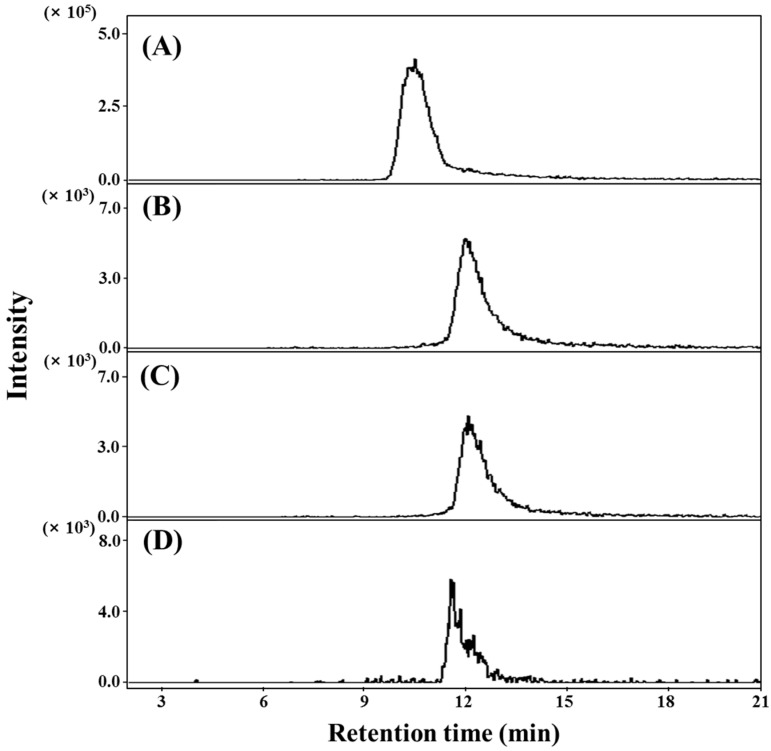
MRM chromatogram for: **3** (**A**); **4** (**B**); **5** (**C**); and **6** (**D**) in *Salicornia herbacea*. Dicaffeoylquinic acid derivatives were analyzed by LC-ESI-MS/MS. Compounds were detected by MRM: *m/z* 517.0 [M − H]^–^ → 355.2 for **3**; *m/z* 531.0 [M − H]^–^ → 161.0 for **4**, *m/z* 531.0 [M − H]^–^ → 161.0 for **5**, and *m/z* 533.0 [M − H]^–^ → 184.2 for **6**. Detailed procedures for LC-ESI-MS/MS analysis have been described in Materials and Methods.

In this study, dicaffeoylquinic acid derivatives **3**–**6** were isolated from the aerial parts of *S. herbacae*. Of these, compounds **4**–**6** were in methyl-esterified forms, suggesting that these compounds are esterified with MeOH under acidic conditions during extraction and purification. Therefore, to confirm the presence of dicaffeoylquinic acid derivatives **3**–**6** including the methyl-esterified derivatives **4**–**6** as native compounds in *S. herbacae*, the EtOAc fraction obtained after the ethanol (EtOH) extraction of the aerial parts of *S. herbacae* was analyzed by selective multiple reaction monitoring (MRM) detection and high performance liquid chromatography/electrospray ionization tandem mass spectrometer (HPLC-ESI/MS). The dicaffeoylquinic acid derivatives isolated in this study were used as external standards. Compounds **3**–**6** were detected at *t*_R_ 10.5, 12.3, 12.5, and 11.8 min on the MRM chromatogram (Figure 2). These data were in agreement with the retention times of the compounds (**3**–**6**). The compounds (**3**–**6**) were also quantitated by selective MRM detection and MS/MS. The external calibration curve for each compound was linear (R^2^ > 0.99) and the recovery rate ranged from 97.0% to 105.5%. Among the dicaffeoylquinic acid derivatives, **3** (75.6 ± 2.3 mg/100 g fresh wt.) was most abundantly found in the aerial part of *S. herbacae*. The other compounds, **4** (69.3 ± 1.4 μg/100 g fresh wt.), **5** (71.9 ± 1.9 μg/100 g fresh wt.), and **6** (171.9 ± 1.5 μg/100 g fresh wt.), were present in smaller amounts in the aerial part of this plant compared to **3**. These results confirm that dicaffeoylquinic acids (**3**–**6**) are unambiguously present in *S. herbacae*.

### 2.3. DPPH Radical-Scavenging Activity of the Isolated Compounds

**Figure 3:**
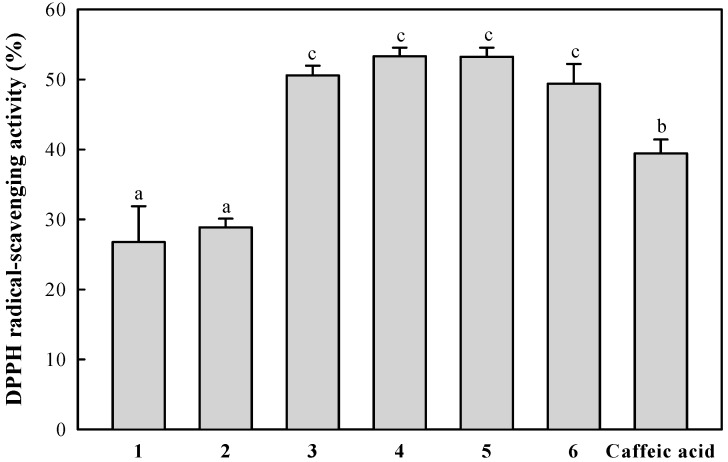
DPPH radical-scavenging activity of the isolated compounds. Each value is the mean ± standard deviation (SD) of three experiments. ^a–c^ Results with a different letter differ significantly (*p* < 0.05).

The radical-scavenging activities of the isolated compounds (final concentration, 10 μM) were evaluated using the DPPH radical. As shown in Figure 3, dicaffeoylquinic acid derivatives **3**–**6**, which contain two catechol groups in the partial structure, showed significantly higher DPPH radical-scavenging activity than the monocaffeoylquinic acid derivatives **1** and **2** as well as caffeic acid, which contains a catechol group. The radical-scavenging activities of dicaffeoylquinic acid derivatives **3**–**6** did not significantly differ (*p* < 0.05), regardless of structural differences like the presence or absence of the olefinic double bond or the binding position of the caffeic and dihydrocaffeic acids to quinic acid. It was reported previously that the catechol structure in phenolic compounds is an important factor for the radical-scavenging effect. These results indicate that the catechol group may be the main contributor to the radical-scavenging activities of the isolated compounds. This pattern agrees with similar results for the DPPH radical-scavenging activities of dicaffeoylquinic acid derivatives isolated from this plant reported in our previous study [19].

### 2.4. Inhibitory Effect of the Isolated Compounds on Copper Ion-Induced Rat Plasma Oxidation

**Figure 4:**
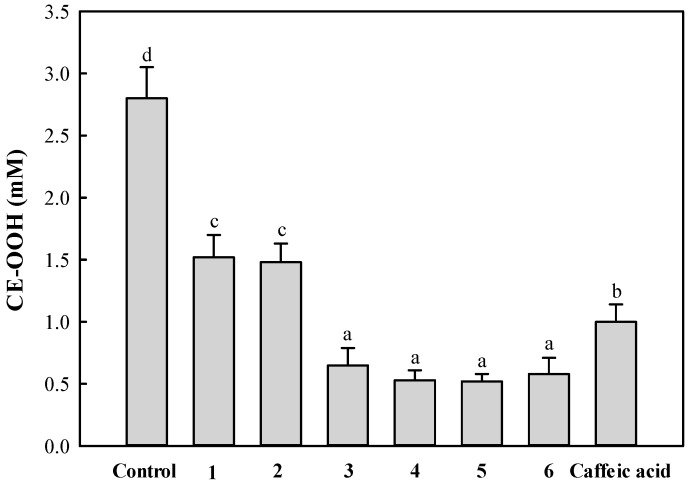
Inhibitory effect against CE-OOH formation by the isolated compounds during copper ion-induced oxidation of diluted rat blood plasma. Rat plasma was diluted four-fold with PBS (pH 7.4) and incubated with 100 μM CuSO_4_ at 37 °C for 7 h. Each compound was added to the rat plasma solution at a final concentration of 10 μM. Each value is the mean ± SD of three experiments. ^a^^–d^ Results with a different letter differ significantly (*p* < 0.05).

Cholesteryl ester hydroperoxide (CE-OOH) produced by oxidation in healthy human plasma is present at a concentration of ca. 3 nM [21]. CE-OOH accumulates in atherosclerotic plaques with the progression of lesion development [22,23]. For this reason, the compound has been used as an index of lipid peroxidation to evaluate the inhibitory effect of antioxidants on lipids oxidized in blood plasma. Therefore, the antioxidant activity of the isolated compounds **1**–**6** (final concentration, 10 μM) in the copper ion induced-blood plasma oxidation system was examined by measuring the CE-OOH content. As shown in Figure 4, caffeoylquinic acid derivatives **1**–**6** considerably inhibited CE-OOH formation when compared to the control (no external addition of antioxidant). In particular, the dicaffeoylquinic acid derivatives **3**–**6** showed a relatively higher ability at inhibiting CE-OOH formation than monocaffeoylquinic acid derivatives **1** and **2** and caffeic acid. This pattern is in good agreement with the results from measuring DPPH radical scavenging.

In this study, four new dicaffeoylquinic acid derivatives isolated from the EtOAc layer of *S. herbacea* were determined to be 3-caffeoyl-5-dihydroquinic acid, 3-caffeoyl-5-dihydrocaffeoylquinic acid methyl ester, 3-caffeoyl-4-dihydrocaffeoylquinic acid methyl ester, and 3,5-dihydrocaffeoylquinic acid methyl ester (Figure 1). In addition, two known compounds, 3-caffeoylquinic acid and 3-caffeoylquinic acid methyl ester, were isolated and identified. To the best of our knowledge, these compounds were identified here for the first time in this plant.

Caffeoylquinic acid derivatives including dicaffeoylquinic acid analogues have been reported to show various biological effects, including antioxidant [19,24], anticancer [25], and anti-inflammatory [26] activities. In this study, the results of the antioxidative evaluation indicated that the caffeoylquinic acid derivatives **1**–**6** significantly scavenged DPPH radicals and inhibited CE-OOH formation during rat blood plasma oxidation induced by copper ions. It is well known that the catechol group has high free radical-scavenging and metal-chelating effects [27,28]. In addition, five other dicaffeoylquinic acid derivatives from the same plant and their high antioxidative activities were reported in our previous study [19]. In this study, we found that DPPH radical-scavenging activities and metal-chelating effects of caffeoylquinic acid derivatives were proportionally correlated with the number of catechol group. Our present and previous observations indicate that the caffeoylquinic acid derivatives **1**–**6** containing a catechol group may act as excellent radical scavengers and metal-chelating agents. In addition, various (di)caffeoylquinic acid derivatives may be abundant in glasswort [29]. These results indicate that the high antioxidative activity of *S. herbacea* may be influenced by various caffeoylquinic acid derivatives.

## 3. Experimental Section

### 3.1. General Experimental Procedures

NMR was recorded on a ^unity^INOVA 500 spectrometer (Varian, Walnut Creek, CA, USA). Mass spectra were acquired on a hybrid SYNAPT G2 (Waters, Cambridge, UK), which was equipped with an electrospray ionization source. Data acquisition took place over the mass range of *m/z* 50 to *m/z* 1200 for MS mode. The sample was introduced into the ESI source at a constant flow rate of 20 µL/min using an external syringe pump (Harvard 11Plus). Thin-layer chromatography (TLC) was carried out using silica gel TLC plates (silica gel 60 F254, 0.25 mm thickness, Darmstadt, Germany) and the fractions were visualized by UV and 1% cerium (IV) sulfate ethanol solution spray. Silica gel column (2.5 cm × 50 cm, Kieselgel 60, 70–230 mesh, Merck, Kenilworth, NJ, USA) and Sephadex LH-20 column (3.5 cm × 55 cm, 25–100 mesh, GE Healthcare Bio-Sciences AB, Uppsala, Sweden) were used for column chromatography. Fractions were purified by HPLC equipped with a Shim-pack Prep-ODS (H) Kit (5 μm, 20 mm × 250 mm; Shimadzu, Kyoto, Japan). The flow rate was 9.9 mL/min, and eluents were monitored at 254 nm.

### 3.2. Materials and Chemicals

Aerial parts of glasswort were collected in June from Younggwang County, located on the southwestern coast of Korea [19]. A voucher sample has been deposited in the warm-temperate forest arboretum located in Bogil Island, Chonnam National University. Solvents used for analyses were of HPLC grade and were purchased from Fisher Scientific Korea. Methanol-*d*_4_ (CD_3_OD) was obtained from Merck. Trifluoroacetic acid, DPPH, caffeic acid, and chlorogenic acid were purchased from Sigma-Aldrich Chemical Co. (St. Louis, MO, USA). All other chemicals and reagents used in this study were of analytical grade.

### 3.3. Extraction and Partition

The MeOH extraction from glasswort and its partition has been reported in our previous study [19]. Briefly, glasswort (8 kg) was homogenized with MeOH (13 L, 2 times). After extraction at room temperature for 24 h, the mixture was filtered through No. 2 filter paper (Whatman International) and concentrated by vacuum evaporation at 38 °C. The MeOH extracts (417.3 g) were suspended in H_2_O (3 L) and partitioned with *n*-hexane (3 L, three times), chloroform (CHCl_3_, 3 L, three times), EtOAc (3 L, three times), and water-saturated *n*-butanol (3 L, three times). Each fraction was evaporated in vacuo at 38 °C.

### 3.4. Isolation of the EtOAc Fraction

The EtOAc layer (6.4 g) was fractionated by silica gel column (2.5 cm × 50 cm) chromatography, eluted with a step-wise system using CHCl_3_/EtOAc/MeOH (10:0:0, 8:2:0, 6:4:0, 4:6:0, 2:8:0, 0:10:0, 0:8:2, 0:6:4, 0:4:6, 0:2:8, 0:0:10, *v*/*v*/*v*, each step 370 mL) and separated into 12 fractions (EA-ET) based on the *R_f_* patterns of spots detected after TLC analysis. Fraction EJ showed relatively higher DPPH radical-scavenging activity than the other fractions. Therefore, fraction EJ (3.4 g, 100% MeOH eluates) was fractionated on Sephadex LH-20 column (3.5 cm × 55 cm) with MeOH (1.5 L) to yield 13 fractions (EJ1–13). Fractions EJ5 (446.3 mg, elution volume/total volume, Ve/Vt, 0.55–0.67) was purified by a linear gradient of 25% MeOH (pH 2.65 by TFA, eluent A) and 60% MeOH (eluent B), starting with 100% A, increasing to 25% B for 5 min, holding at 25% B over 10 min, increasing to 100% B until 35 min, and holding at 100% B for 10 min (ODS-1) to give 16 subfractions (EJ5-1–16). Compound **6** (3.3 mg) from subfraction EJ5-2 (*t*_R_ 20.33 min, 8.9 mg) and **3** (110.1 mg) from subfraction EJ5-3 (*t*_R_ 20.91 min, 136.5 mg) were isolated by further purification with the same HPLC condition. Fraction EJ6 (1055.2 mg, Ve/Vt 0.68–0.83) was purified by the same HPLC condition described above to give 10 subfractions (EJ6-1–10). Subfractions EJ6-4 (*t*_R_ 23.2 min, 86.0 mg) and EJ6-7 (*t_R_* 31.2 min, 68.6 mg) were further purified using 40% MeOH (pH 2.65 by TFA) and 45% MeOH (pH 2.65 by TFA), respectively, by an isocratic system. Compounds **1** (*t*_R_ 7.3 min, 1.3 mg), **2** (*t_R_* 9.30 min, 2.4 mg), **3** (*t*_R_ 17.0 min, 11.7 mg), and **4** (*t_R_* 33.7 min, 10.1 mg) from subfraction EJ6-4 and **5** (*t_R_* 28.9 min, 1.3 mg) from EJ6-7 were isolated.

*Compound* **1** (white amorphous powder): ^1^H-NMR (500 MHz, CD_3_OD) δ 2.01–2.20 (4H, m, H-2, 6), 5.33 (1H, m, H-3), 3.65 (1H, dd, *J* = 8.0, 3.5 Hz, H-4), 4.13 (1H, m, H-5), 7.04 (1H, br. s, H-2′), 6.77 (1H, d, *J* = 7.8 Hz, H-5′), 6.94 (1H, br. d, *J* = 7.8 Hz, H-6′), 7.68 (1H, d, *J* = 15.5 Hz, H-7′), 6.31 (1H, d, *J* = 15.5 Hz, H-8′); ESI-MS (negative) *m/z* 353.2 [M − H]^–^.

*Compound* **2** (white amorphous powder): ^1^H-NMR (500 MHz, CD_3_OD) δ 1.98–2.21 (4H, m, H-2, 6), 5.35 (1H, m, H-3), 3.68 (1H, dd, *J* = 8.0, 3.5 Hz, H-4), 4.11 (1H, m, H-3), 3.72 (3H, s, -OCH_3_), 7.04 (1H, d, *J* = 2.0 Hz, H-2′), 6.77 (1H, d, *J* = 8.0 Hz, H-5′), 6.94 (1H, dd, *J* = 8.0, 2.0 Hz, H-6′), 7.68 (1H, d, *J* = 15.5 Hz, H-7′), 6.31 (1H, d, *J* = 15.5 Hz, H-8'); ESI-MS (negative) *m/z* 367.2 [M − H]^–^.

*Compound* **3** (white amorphous powder): ${\left[\alpha \right]}_{D}^{25}$ –2.3 (*c* = 0.1, MeOH); ^1^H- and ^13^C-NMR data are listed in Table 1 and Table 2; HRESI-MS (negative) *m/z* 517.1334 [M − H]^–^ (calculated for C_25_H_25_O_12_, *m/z* 517.1346, −1.2 mDa).

*Compound* **4** (white amorphous powder): ${\left[\alpha \right]}_{D}^{25}$ –12.8 (*c* = 0.1, MeOH); ^1^H- and ^13^C-NMR data are shown in Table 1 and Table 2; HRESI-MS (negative) *m/z* 531.1500 [M − H]^–^ (calculated for C_26_H_27_O_12_, *m/z* 531.1503, −0.3 mDa).

*Compound* **5** (white amorphous powder): ${\left[\alpha \right]}_{D}^{25}$ –13.6 (*c* = 0.1, MeOH); ^1^H- and ^13^C-NMR data are shown in Table 1 and Table 2; HRESI-MS (negative) *m/z* 531.1507 [M − H]^–^ (calculated for C_26_H_27_O_12_, *m/z* 531.1503, +0.4 mDa).

*Compound* **6** (white amorphous powder): ${\left[\alpha \right]}_{D}^{25}$ –12.4 (*c* = 0.1, MeOH); ^1^H- and ^13^C-NMR data are shown in Table 1 and Table 2; HRESI-MS (negative) *m/z* 533.1655 [M − H]^–^ (calculated for C_26_H_29_O_12_, *m/z* 533.1659, −0.4 mDa).

### 3.5. HPLC ESI MS/MS Analysis of Four Dicaffeoylquinic Acid Derivatives Identified in S. herbacea

Fresh aerial components of *S. herbacea* (10 g) were homogenized in EtOH (150 mL). The mixture was filtered under vacuum through No. 2 filter paper (Whatman). The residue was homogenized in 80% EtOH (150 mL) and filtered through No. 2 filter paper. The EtOH and 80% EtOH solutions were combined and concentrated under a vacuum at 38 °C. The extracts were suspended in distilled water (100 mL), the pH was adjusted to 2.6 with 1.0 M HCl solution, and partitioning was performed with *n*-hexane and EtOAc (100 mL, three times). The EtOAc fraction was evaporated under a vacuum at 38 °C and dissolved in 100% MeOH (10 mL). The EtOAc fraction was analyzed using a LC-ESI/MS (Shimadzu). The isolated compounds (**3**–**6**) were separated under the chosen HPLC conditions [column, MG III (C18, 3 *µ*m, 3.0 mm × 100 mm) (Shiseido, Tokyo, Japan); column temperature, 35 °C; flow rate, 0.3 mL/min]. The sample was eluted using a gradient system of H_2_O (solvent A) to acetonitrile (solvent B) (both containing 0.1% formic acid), starting with 10% B for 1 min, increasing to 23% B for 2 min, holding at 23% A for 5 min, increasing to 33% A for 7.5 min, holding at 33% A for 18 min, increasing to 90% A for 18.5 min, and holding at 90% A for 23 min. The mass spectrometer was subsequently set up for MRM with a dwell time of 0.1 s per transition to monitor the dicaffeoylquinic acid derivatives: *m/z* 517.0 [M – H]^–^ → 355.2 for **3**; *m/z* 531.0 [M – H]^–^ → 161.0 for **4**, *m/z* 531.0 [M – H]^–^ → 161.0 for **5**, and *m/z* 533.0 [M – H]^–^ → 184.2 for **6**. The optimal MS conditions for **3**–**6** were employed: ESI source voltage 3.5 kV; detector voltage 45 V; heat block temperature, 400 °C; desolvation line temperature, 250 °C. Nebulizing gas and drying gas flows were 3.0 L/min and 15.0 L/min, respectively. Argon was used as the collision gas at a pressure of 230 kPa. The optimized collision energy for **3**, **4**, **5**, and **6** were 24, 37, 33, and 31 V, respectively.

The contents of **3**–**6** in *S. herbacea* were quantitatively analyzed by LC-ESI-MS/MS. Sample and standard solutions were prepared just before analysis. The calibration curves (*n* = 6) were constructed using compounds **3**–**6** (0.1–50 ng) isolated from this plant. Accuracy and reproducibility were evaluated using the standard spike method. External standards of **3**–**6** were added to aliquots of the aerial parts of *S. herbacea* at three concentrations to determine the precision. The quantification and quantitation of **3**–**6** in the aerial parts of *S. herbacea* were performed in triplicate. 

### 3.6. Assay of DPPH Radical-Scavenging

The assay for purification of the antioxidative compounds was conducted by TLC using the method described by Takao et al. [30], with slight modifications. Briefly, all fractions obtained in the purification process were spotted on a silica gel TLC and developed using a mixture of *n*-BuOH/acetic acid/H_2_O = 4:1:1 (*v*/*v*/*v*). The developed TLC was sprayed with 200 μM DPPH free radical EtOH solution and the decolorized spots were considered to be antioxidative compounds.

The free radical-scavenging activities of the isolated compounds, with caffeic acid as a positive control, were also evaluated by ODS-HPLC analysis as in previous research but with slight modifications [31]. Briefly, an ethanol solution (50 μL) of each compound (final concentration, 10 μM) was mixed with DPPH radical ethanol solution (150 μL; final concentration, 100 μM). After standing for 20 min in the dark, the mixture was transferred to a HPLC system connected to a TSK-gel Octyl-80Ts column (5 μm, 4.6 mm × 25 cm; Tosoh, Tokyo, Japan). The elution was carried out with an isocratic system of acetonitrile/H_2_O = 60:40 (*v*/*v*). The flow rate was 1.0 mL/min and the remaining DPPH radical was monitored at 517 nm. The DPPH radical-scavenging activities of each sample were determined as the percentage decrease compared to the peak area of the DPPH radical in a blank sample.

### 3.7. Determination of the Inhibitory Effect of the Isolated Compounds against Copper Ion-Induced Rat Plasma Oxidation

The antioxidative activities of the isolated compounds were evaluated by measuring their inhibitory effects against CE-OOH formation in copper ion-induced oxidation of diluted rat blood plasma [19]. Sprague-Dawley rats (male, 6 weeks age, 180–200 g) (Samtako Bio Korea) were kept at 23 °C under a 12 h dark/light cycle and fasted for 12–15 h prior to blood collection. After anesthesia with diethyl ether, the abdomen wall was opened, and blood was collected from the abdominal aorta into heparinized tubes. Rat plasma was isolated by centrifugation (1500× *g*) at 4 °C for 20 min and used immediately for experiments or stored at –40 °C for no longer than 1 week. All experimental procedures were approved by the Institutional Animal Care and Use Committee of Chonnam National University (no. CNU IACUC-YB-R-2013-4). The plasma was diluted four-fold in PBS buffer (pH 7.4) and the diluted plasma was mixed with an EtOH solution (final 1%) of the isolated compounds (final concentration, 10 μM). The mixture was oxidized with the addition of 0.1 mL of CuSO_4_ PBS solution (final concentration, 100 μM). After incubation at 37 °C for 7 h with continuous shaking, an aliquot (100 μL) was mixed with 3 mL of MeOH containing 2.5 mM 2,6-di-*tert*-butyl-4-methylphenol and partitioned with *n*-hexane (3 mL, 2 times). The upper layer (*n*-hexane) was concentrated under a vacuum and then dissolved with 100 μL of MeOH/CHCl_3_ (95:5, *v*/*v*). The dissolved solution was transferred to a HPLC system (Shimadzu) equipped with a TSK-gel Octyl-80Ts column (Tosoh). Elution was performed with an isocratic system of MeOH/H_2_O (97:3, *v*/*v*). The flow rate was 1.0 mL/min, and the CE-OOH produced was monitored at 235 nm. The concentration of CE-OOH was calculated from a standard curve for cholesteryl linoleate hydroperoxide. Detailed procedures for preparation of the cholesteryl linoleate hydroperoxide standard have been published elsewhere [32].

### 3.8. Statistical Analysis

The data for the antioxidative activity of the isolated compounds were expressed as mean ± SD using the Statistical Package for Social Sciences 19.0 package programs (IBM, Armonk, NY, USA). Statistical differences were measured by one-way ANOVA followed by Duncan’s multiple comparison test. Significant difference was set at *p* < 0.05.

## 4. Conclusions

Four new dicaffeoylquinic acid derivatives and two known compounds were isolated from the aerial parts of *Salicornia herbacea*. In addition, the presence of dicaffeoylquinic acid derivatives in this plant was reconfirmed by LC-ESI-MS/MS analysis. The caffeoylquinic acid derivatives **1**–**6** scavenged DPPH radicals and inhibited the CE-OOH formation in copper ions-induced rat blood plasma oxidation. *S. herbacea* can be viewed as a promising health-promoting vegetable and medical source because of the high antioxidative activity of the various caffeoylquinic acid derivatives found in the plant. 

## Figures and Tables

**Table 1:** ^1^H-NMR (500 MHz) data for **3**–**6** in CD_3_OD.

Position	δ_H_ (int., multi., *J* in Hz)			
	3	4	5	6
2ax	2.27 (1H, dd, 14.0, 4.0)	2.26 (1H, dd, 14.0, 4.0)	2.24 (1H, dd, 12.0, 2.5)	2.19 (1H, dd, 14.0, 3.5)
2eq	2.14 (1H, dd, 14.0, 7.5)	2.13 (1H, dd, 14.0, 7.5)	2.08 (1H, dd, 12.0, 7.5)	2.05 (1H, dd, 14.0, 7.5)
3	5.37 (1H, m)	5.32 (1H, m)	5.36 (1H, m)	5.18 (1H, m) ^a^
4	3.88 (1H, dd, 7.5, 3.0)	3.86 (1H, dd, 7.5, 3.0)	5.02 (1H, dd, 7.5, 3.5)	3.79 (1H, dd, 7.2, 3.0)
5	5.29 (1H, m)	5.25 (1H, m)	4.26 (1H, m)	5.18 (1H, m) ^a^
6ax	2.11 (1H, br. d, 5.5)	2.10 (2H, br. d, 5.5)	2.15 (1H, dt, 9.0, 2.0)	2.06 (2H, br. d, 5.0)
6eq	2.11 (1H, br. d, 5.5)		2.09 (1H, dt, 12.0, 2.0)	
-OCH_3_	-	3.72 (3H, s)	3.69 (3H, s)	3.72 (3H, s)
2′	7.06 (1H, d, 1.8)	7.06 (1H, d, 2.0)	7.03 (1H, d, 2.0)	6.66 (1H, d, 2.4)
5′	6.77 (1H, d, 7.8)	6.77 (1H, d, 8.0)	6.78 (1H, d, 8.0)	6.67 (1H, d, 8.4)
6′	6.96 (1H, dd, 8.0, 1.8)	6.96 (1H, dd, 8.0, 2.0)	6.95 (1H, dd, 8.0, 2.0)	6.55 (1H, dd, 8.4, 2.4)
7′	7.61 (1H, d, 15.5)	7.61 (1H, d, 16.0)	7.50 (1H, d, 15.5)	2.79 (2H, m)
8′	6.34 (1H, d, 15.5)	6.32 (1H, d, 16.0)	6.16 (1H, d, 15.5)	2.60 (2H, m)
2″	6.66 (1H, br. s)	6.64 (1H, d, 2.0)	6.62 (1H, d, 2.0)	6.63 (1H, d, 2.1)
5″	6.67 (1H, d, 8.0)	6.66 (1H, d, 8.0)	6.63 (1H, d, 8.0)	6.65 (1H, d, 8.4)
6″	6.54 (1H, dd, 8.0, 2.0)	6.53 (1H, dd, 8.0, 2.0)	6.48 (1H, dd, 8.0, 2.0)	6.52 (1H, dd, 8.4, 2.1)
7″	2.79 (2H, m)	2.77 (2H, m)	2.76 (2H, m)	2.76 (2H, m)
8″	2.60 (2H, m)	2.60 (2H, m)	2.61 (2H, m)	2.56 (2H, m)

^a^ The chemical shifts of H-3 and H-5 overlapped.

**Table 2:** ^13^C-NMR (125 MHz) data for **3**–**6** in CD_3_OD.

Position	3	4	5	6
1	75.6	75.6	75.0	75.0
2	37.8	37.5	38.4	37.4
3	72.7	74.9	75.4	72.2 ^a^
4	70.8	72.6	74.8	70.1 ^a^
5	72.3	72.2	69.2	72.2
6	36.2	35.9	37.4	35.8
7	175.9	175.9	175.2	175.6
-OCH_3_	-	53.1	53.2	3.1
1′	128.1	128.0	127.6	133.6
2′	115.3	115.2	115.3	116.6
3′	146.9	147.0	147.0	146.3
4′	147.2	147.3	147.9	144.9
5′	116.6	116.6	116.7	116.5
6′	123.1	123.2	123.2	120.9
7′	149.6	149.7	150.0	37.9
8′	115.7	115.6	114.6	31.4
9′	169.0	169.0	167.8	174.6
1″	133.7	133.5	133.5	133.3
2″	116.6	116.6	116.5	116.6
3″	146.3	146.4	146.4	146.3
4″	144.8	144.8	144.8	144.9
5″	116.5	116.5	116.5	116.5
6″	120.7	120.7	120.6	120.9
7″	37.5	37.5	37.4	37.5
8″	31.4	31.5	31.7	31.6
9″	174.2	174.0	174.2	173.9

^a^ The chemical shifts of C-3 and C-5 overlapped.
